# Role of Routine Blood Parameters in Predicting Mortality Among Surgical Patients With Sepsis

**DOI:** 10.7759/cureus.37413

**Published:** 2023-04-10

**Authors:** Srishti Dixit, Jainendra K Arora, Rakesh Kumar, Rashmi Arora

**Affiliations:** 1 General Surgery, Vardhman Mahavir Medical College and Safdarjung Hospital, New Delhi, IND; 2 Cancer Surgery, Vardhman Mahavir Medical College and Safdarjung Hospital, New Delhi, IND; 3 Pathology, Vardhman Mahavir Medical College and Safdarjung Hospital, New Delhi, IND

**Keywords:** mean platelet volume (mpv), platelet distribution width, platelet count (plt), red cell distribution width (rdw), sepsis, mortality

## Abstract

Background: Outcome prediction for surgical patients with sepsis may be conducive to early aggressive interventions. In several studies, changes in the level of numerous biomarkers like red cell distribution width (RDW), platelet count (PC), mean platelet volume (MPV), and platelet distribution width (PDW) have been demonstrated to be associated with mortality in critically ill patients. We aimed at investigating the prognostic significance of dynamic changes in RDW, PC, MPV, and PDW in surgical patients with sepsis.

Methods: We prospectively enrolled 110 surgical patients of sepsis in our study admitted to the surgical ward and ICU. We measured RDW, PC, MPV, and PDW on days 1, day 4, and day 8. Receiver operating characteristics (ROC) were generated for prognostic validation of these parameters and mortality in surgical patients with sepsis.

Results: We found that higher RDW and PDW on day 1 among non-survivors as compared to survivors on day 1 were significantly associated with mortality. ROC curves showed that RDW and PDW on day 1 could be used to predict mortality in surgical patients with sepsis and it was dynamic changes in PC on day 4 and day 8 along with a change in MPV on day 8, which was significantly associated with mortality.

Conclusion: The major findings of our study were baseline value of RDW and PDW on day 1 and continuous decrease in PC and increase in MPV over one week were significantly associated with mortality. So, it is better to monitor dynamic changes in PC and MPV in combination with baseline RDW and PDW. So, these parameters can be promising markers to assess prognosis in surgical patients with sepsis.

## Introduction

Dysregulated host response to infection causes sepsis. Sepsis and septic shock are major healthcare problems. In clinical practice, patients with sepsis requiring immediate treatment can be identified using prognostic sepsis scores like Sequential Organ Failure Assessment (SOFA) score, and Acute Physiology and Chronic Health Evaluation (APACHE) II score. In recent years, red cell distribution width (RDW) [1,2], platelet count (PC) [3], mean platelet volume (MPV) [4], and platelet distribution width (PDW) [5,6] are associated with adverse prognosis in various non-infectious, infectious diseases and critically ill ICU patients. The exact pathophysiological mechanism behind this association is not clearly understood but systemic factors that alter erythrocyte and platelet homeostases such as inflammation and oxidative stress seem to play an important role [7,8].

RDW is a standard parameter of the complete blood count (CBC) and indicates variability in RBC size; the proportional variation in mean corpuscular volume (MCV) is RDW. MPV is the average size of platelets in a blood sample. PC is a standard haematological parameter routinely measured. PDW measures variation in platelet size, which is an indicator of active platelet release. All these four parameters are measured as part of an automated CBC panel and so are easily accessible and inexpensive.

Previous studies have mainly focused on the study of these parameters in non-surgical critically ill patients as a single measurement and the significance of the dynamic changes in their values in surgical patients with sepsis is yet to be established. So, this study was done to predict mortality in surgical patients with sepsis using RDW, PC, MPV, and PDW.

## Materials and methods

The study was a hospital-based prospective observational cohort study conducted from October 2020 to March 2022 after obtaining approval from the Institutional Ethics Committee of Vardhman Mahavir Medical College and Safdarjung Hospital, New Delhi, India (approval number: IEC/VMMC/SJH/Thesis/2020-11/CC-145). One hundred and ten patients admitted with sepsis in the department of surgery and intensive care units of Vardhman Mahavir Medical College and Safdarjung Hospital, New Delhi, India were studied. Written informed consent from the patients or their legal guardians was obtained.

The sample size of 110 patients was chosen based on the study of Kim et al. [9]. Taking their values as a reference, the minimum required sample size with 80% power of the study, 5% level of significance, and 10% sampling error was 110 patients. 

Inclusion and exclusion criteria

Inclusion criteria were: (i) Age above 12 years and (ii) Patients in the surgical ward who met the criteria of sepsis or septic shock. Exclusion criteria were: (i) Blood product transfusion one week before admission, (ii) Patients with a known history of diseases affecting platelet indices (e.g present history of infections like dengue, vasculitis, and other autoimmune disorders., malignancy), (iii) Use of drugs known to change morphology and rheology of RBCs (e.g immunosuppressants, steroids), (iv) Immunocompromised states (e.g. HIV positive, malignancy, chemo or radiotherapy), and (v) Pregnant and lactating women.

Data collection

A total of 110 patients with sepsis who fulfilled the criteria of sepsis and septic shock were included. Sepsis was defined according to standard Sepsis-3 as organ dysfunction identified as an acute change in total SOFA score ≥2 points after infection. Septic shock was defined as criteria for Sepsis-3 and both of the: (i) Persisting hypotension requiring vasopressors to maintain mean arterial pressure (MAP) ≥65 mmHg and (ii) Lactate ≥2 mmol/L

All the patients were managed according to the Surviving Sepsis Campaign 2013 Guidelines. Standardized data collection forms were used to collect the information about demographic, clinical, and laboratory parameters of the study subjects. SOFA was determined using the worst values within 24 hours of admission.

Laboratory measurements

Standardized data collection forms were used to collect information about the demographic, clinical, and laboratory parameters of the study subjects. A five-part automated haematology analyzer measured the CBC and RDW, PC, MPV, and PDW were recorded as part of it. Blood samples were analyzed on day 1, i.e. at initial presentation, at 72 hours (day 4), and after one week, i.e. eighth day of sepsis. Day 1 was considered as the first day that severe sepsis was diagnosed or on admission (baseline values) if the patient was in sepsis when admitted. Day 4 was considered as the day after 72 hours elapsed. Patients were assessed for 30 days mortality as an endpoint. The primary outcome measure was 30-day mortality after admission. We chose to use 30-day mortality as it is an objective, clinically relevant, and well-accepted outcome indicator in studies about emergency and critical care areas and it is less dependent on hospital discharge practices/policies.

Statistical analysis

The presentation of the categorical variables was done in the form of numbers and percentages (%). On the other hand, the quantitative data were presented as the means ± SD and as the median. The association of the variables which were quantitative and not normally distributed in nature was analyzed using Mann-Whitney Test and the Independent t-test was used for the association of normally distributed data with the outcome. The association of the qualitative variables was analyzed using the Chi-Square test. The receiver operating characteristic (ROC) curve was used to find out the cut-off point of RDW, PC, and PDW on day 1, and their changes on day 4 and day 8 for predicting mortality. Multivariate logistic regression was used to find out independent significant risk factors of mortality. The data entry was done in the Microsoft Excel spreadsheet (Microsoft Corporation, Redmond, Washington, United States) and the final analysis was done with IBM SPSS Statistics for Windows, Version 21.0 (Released 2012; IBM Corp., Armonk, New York, United States).

## Results

Baseline characteristics

In the study cohort, most of the patients were in the age group of 31-40 years with a mean age of 41.24 years and 57.27% of patients were male. The acute abdomen was the most common cause of presentation at our institute. When the primary outcome was taken as 30-day mortality, 51.82% of patients succumbed to mortality. Increasing age was found to be a significant risk factor for mortality with a mean age of non-survivors was 46.19 years in our study, which is higher than the age of the survivors. Increasing age was significantly associated with mortality (p-value-0.002), which was 100% in the age group of 61-70 years. Similarly, the mean SOFA score amongst non-survivors was 3.45, which was again higher than that of the survivors and was significantly associated with mortality with a p-value of 0.0002.

**Figure 1:**
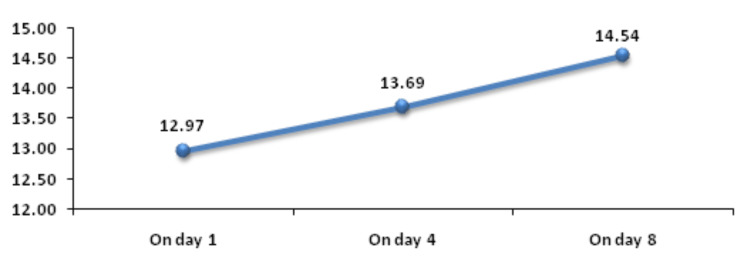
Trend of RDW (%) on day 1, day 4, and day 8 RDW: red cell distribution width

**Figure 2:**
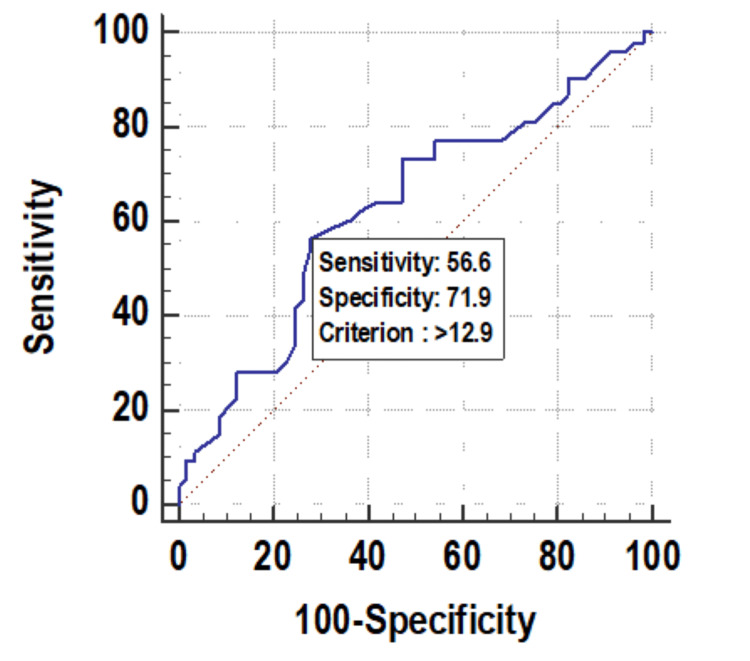
Receiver operating characteristic curve of mean RDW (%) on day 1 RDW: red cell distribution width

**Figure 3:**
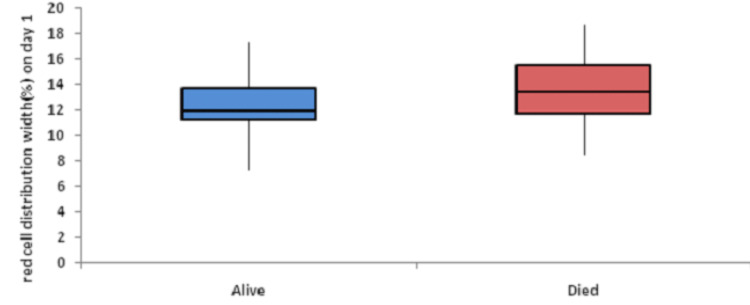
Association of mean RDW (%) on day 1 with outcome using box and whisker plot RDW: red cell distribution width

Baseline RDW was significantly associated with mortality. Figure 1 shows descriptive characteristics of RDW on day 1, day 4, and day 8 and it was found that mean RDW was increasing on day 4 and day 8 with values of 13.69 and 14.54, respectively, when compared to the mean baseline value of 12.97. The ROC curve of mean RDW on day 1 for mortality assessment was drawn and the area under the curve (AUROC) was found to be 0.629, standard error of 0.0539, cut off 12.9, p-value 0.017, 95%CI 0.531-0.719, which was statistically significant with sensitivity and specificity of 56.6 and 71.9, respectively (Figure 2). At the same time, AUROC for change in RDW on day 4 and day 8 as compared to day 1 was not found to be statistically insignificant (p-value >=0.05). Figure 3 shows the association of mean RDW on day 1 with outcome using a box and whisker plot (boxes represent mean +- SD). Mann-Whitney test established a significant association between mean RDW on day 1 and mortality with a p-value of 0.02, but a change in RDW was not found to be associated with mortality.

**Figure 4:**
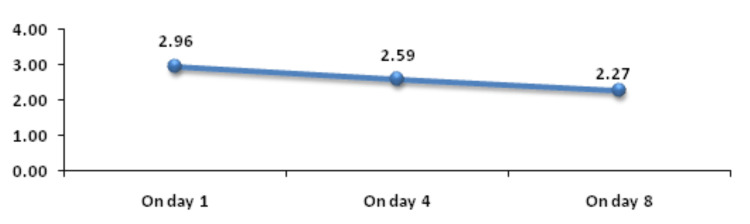
Trend of PC in lakhs (cells/mm3) on day 1, day 4, and day 8 PC: platelet count

**Figure 5:**
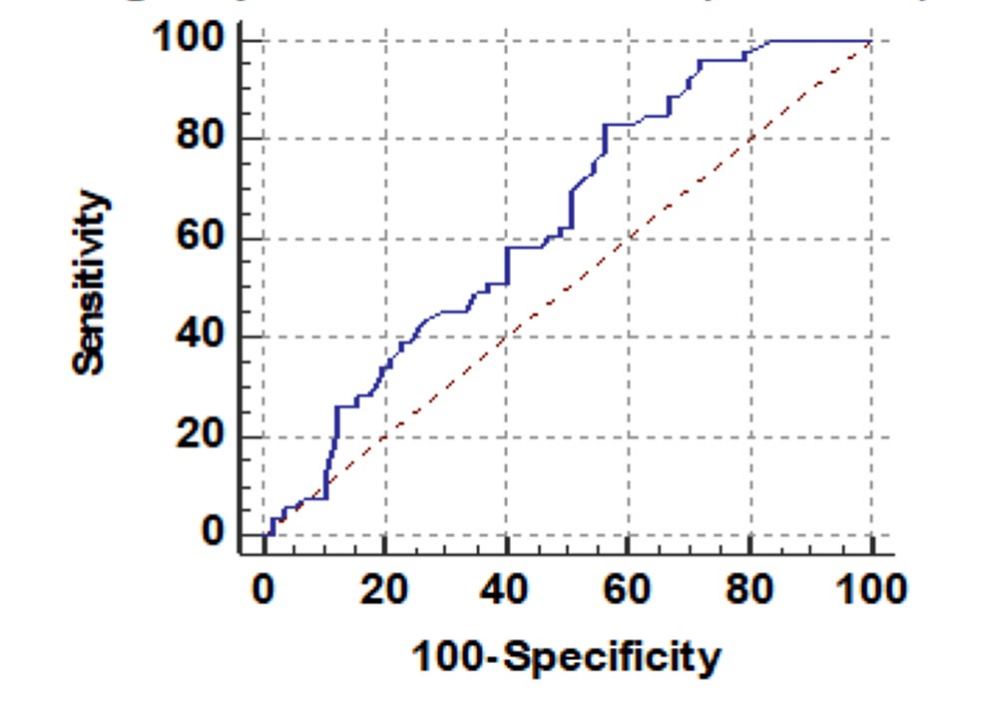
Receiver operating characteristic curve of mean change in PC in lakhs (cells/mm3) on day 4 PC: platelet count

**Figure 6:**
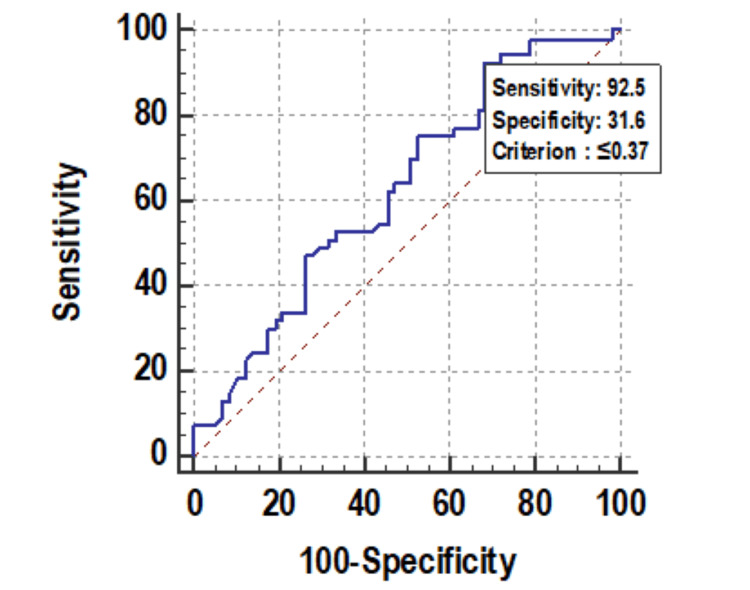
Receiver operating characteristic curve of mean change in PC in lakhs (cells/mm3) on day 8 PC: platelet count

**Figure 7:**
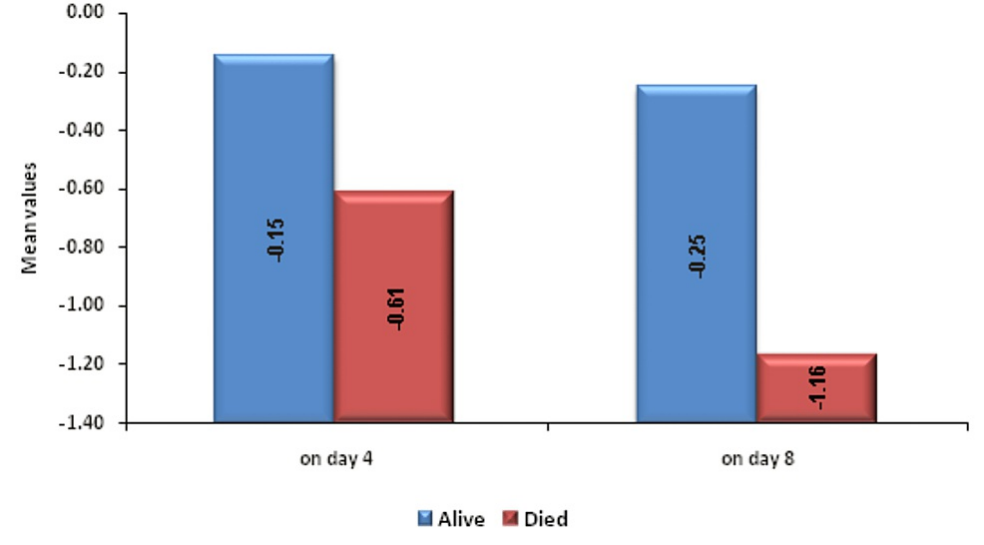
Association between change in mean PC in lakhs (cells/mm3) on day 4 and day 8 with outcome PC: platelet count

Change in PC on day 4 and on day 8 was significantly associated with mortality. Figure 4 shows descriptive characteristics of PC on day 1, day 4, and day 8 and that means PC was decreasing on day 4 and day 8 with values of 2.59 and 2.27, respectively, when compared to the mean baseline value of 2.96. ROC curve of mean change in PC on day 4 and day 8 for mortality assessment was drawn and AUROC was found to be 0.635 (p value-0.0106) for day 4 and 0.628 (p value-0.0165) for day 8, standard error of 0.0539, which was statistically significant (Figures 5, 6). Figure 7 shows an association between change in mean PC on day 4 and day 8 with mortality and it was found that change in PC with decrease as the change was significantly associated with mortality with p-value of 0.012 for day 4 and 0.006 for day 8. At the same time, baseline PC was not significantly associated with mortality with p-value of 0.18.

**Figure 8:**
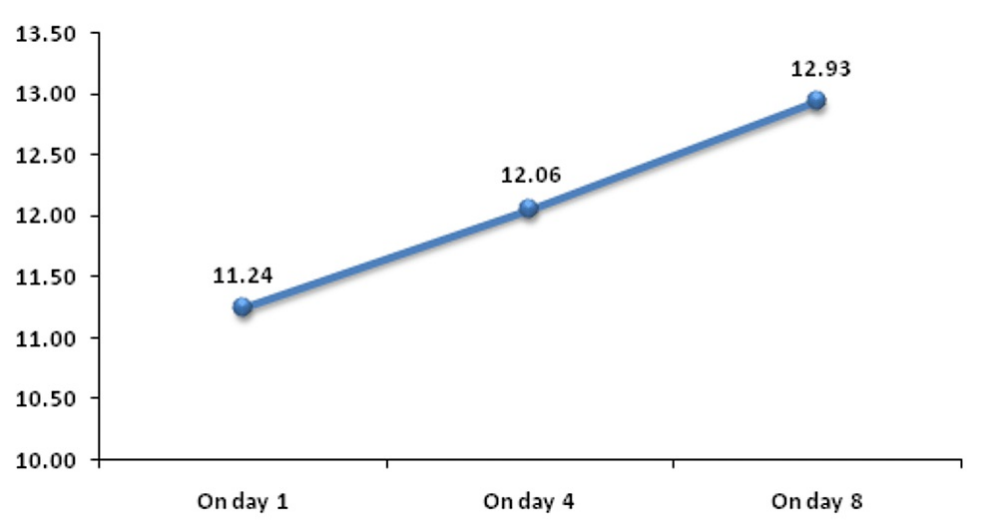
Trend of MPV (cm3) on day 1, day 4, and day 8 MPV: mean platelet volume

**Figure 9:**
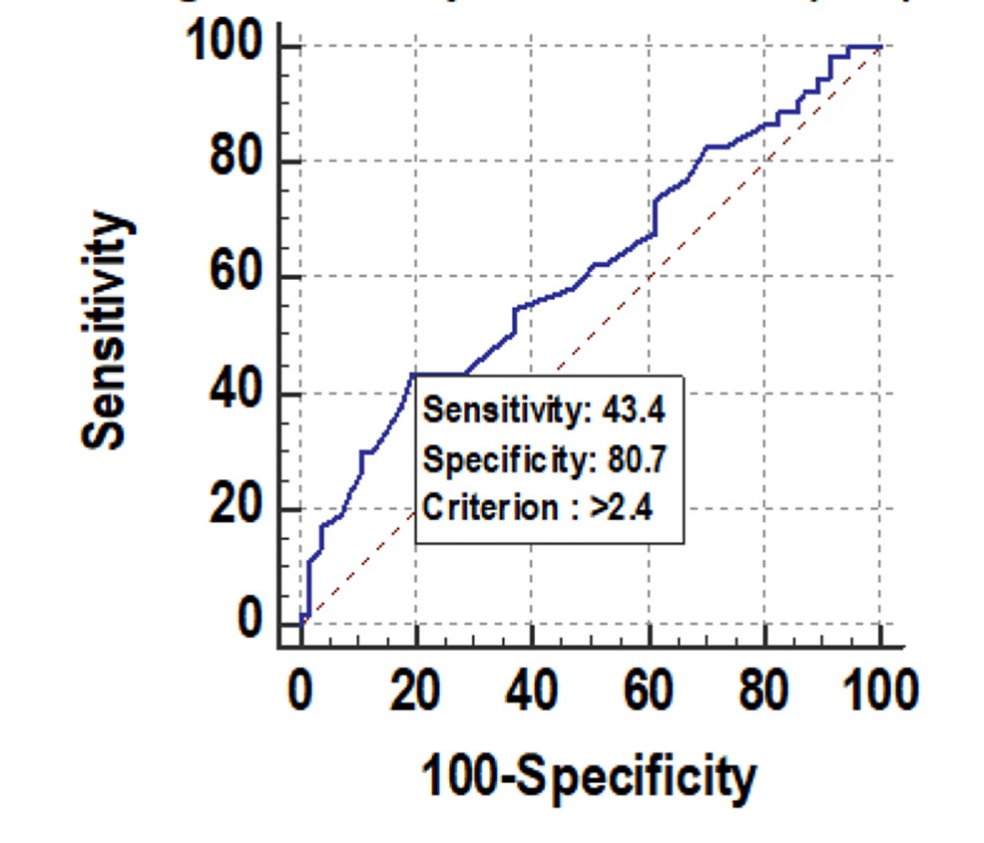
Receiver operating characteristic curve of mean of change in MPV (cm3) on day 8 for mortality assessment MPV: mean platelet volume

**Figure 10:**
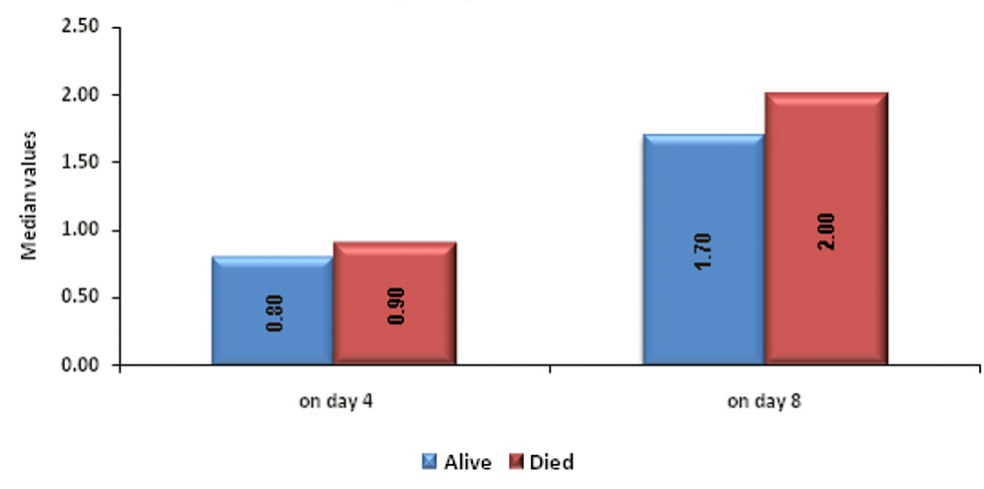
Association between change in MPV (cm3) on day 4 and day 8 with outcome MPV: mean platelet volume

Change in MPV on day 8 was significantly associated with mortality. Figure 8 shows descriptive characteristics of MPV on day 1, day 4, and day 8 and that mean MPV was increasing on day 4 and on day 8 with values of 12.06 and 12.93, respectively, when compared to the mean baseline value of 11.24. ROC curve of mean change in MPV on day 8 for mortality assessment was drawn and AUROC was found to be 0.614, standard error 0.0541, cut off >2.4, p-value 0.0352, 95%CI 0.516-0.705, which is statistically significant (Figure 9) but AUROC for baseline MPV and change in MPV on day 8 was 0.585 and 0.593, respectively, with p-value of 0.128 and 0.08, respectively, which is not statistically significant. A significant association was established using Mann-Whitney test between the mean change in MPV on day 8 from day 1 with an increase as the change (p-value 0.031) but baseline MPV and change within 72 hours, i.e. on day 4, was not significantly associated with mortality with p-value >=0.05 (Figure 10).

**Figure 11:**
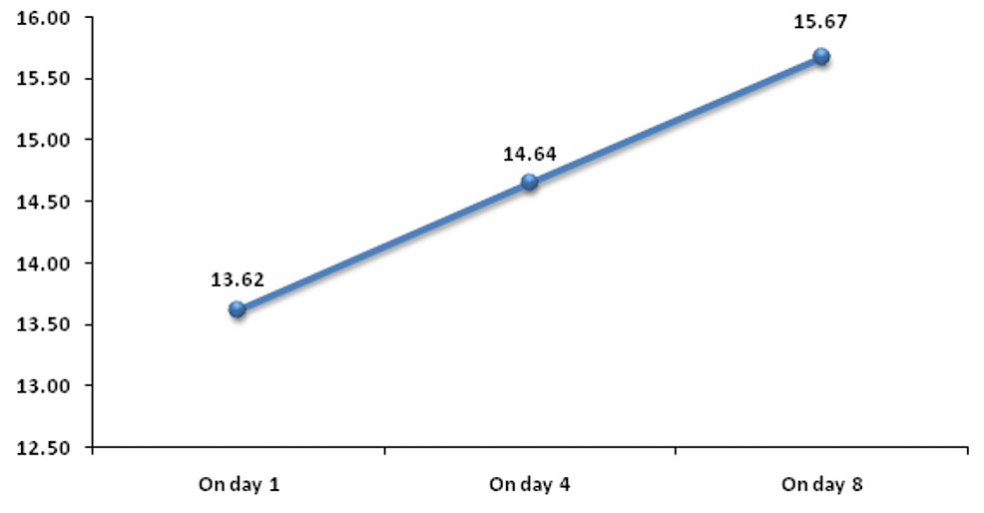
Trend of PDW (%) on day 1, day 4, and day 8 PDW: platelet distribution width

**Figure 12:**
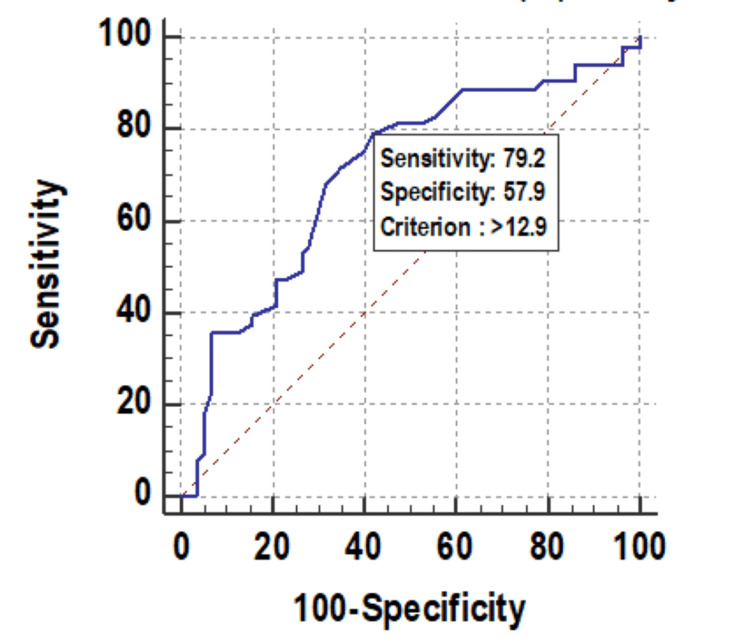
Receiver operating characteristic curve of mean PDW (%) on day 1 for mortality assessment PDW: platelet distribution width

**Figure 13:**
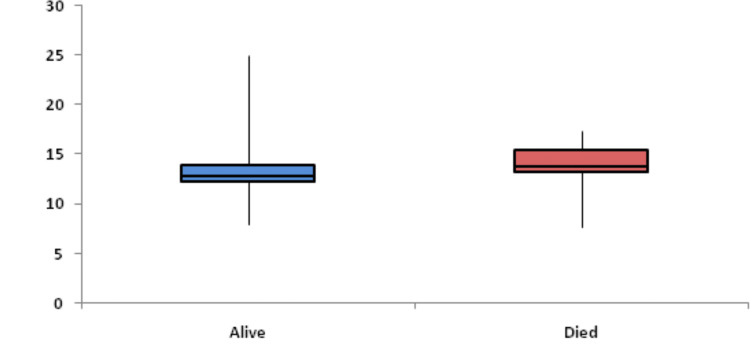
Association of mean PDW (%) on day 1 with outcome using box and whisker plot PDW: platelet distribution width

PDW on day 1 was significantly associated with mortality. Figure 11 shows descriptive characteristics of PDW on day 1, day 4, and day 8 and that mean PDW was increasing on day 4 and on day 8 with values of 13.62 and 14.64, respectively when compared to the mean baseline value of 15.67. ROC curve of mean PDW on day 1 for mortality assessment was drawn and AUROC was found to be 0.703, standard error 0.0509 , cut off > 12.9, p-value 0.001, 95%CI 0.608-0.786, which was statistically significant with sensitivity and specificity of 79.2 and 57.9, respectively (Figure 12). At the same time, AUROC for change in PDW on day 4 and on day 8 from day 1 was not found to be statistically insignificant (p-value >=0.05). Figure 13 shows the association of mean PDW on day 1 with outcome using box and whisker plot (boxes represent mean +- SD). Mann-Whitney test established a significant association between mean PDW on day 1 and mortality with p-value of 0.0002, but change in PDW was not found to be associated with mortality.

## Discussion

RDW, PC, MPV, and PDW are common, inexpensive, easily accessible laboratory parameters in patients of suspected infection. Numerous studies done previously have shown an independent association of these parameters with adverse outcomes in various diseases. This study was done to predict mortality in surgical patients with sepsis. The difference in our study was in the choice of subjects who not only suffered the burden of the disease process but also of the surgical procedure done. Any surgery adds to the stress of the body and brings into a fulminant stress response which is higher than in a medical disease.

The results of our study were comparable to the previous studies. Higher RDW, even if the value lies in the normal range (cut off in our study was 13.5), on day 1 was found to be significantly associated with mortality similar to the study by Jo et al. [10] with comparable AUROCs. The potential mechanism behind this association could be the effect of oxidative sress in sepsis causing alternation in the glycoproteins and ion channels on RBC membrane. We could not establish a significant association between the change in RDW over the first week with mortality as seen in previous studies [11]. This could be due to the other factors that are known to affect RDW like serum iron, vitamin B12, and folic acid levels, which were not taken into account in our study.

When platelet indices were studied, it was found in our study that dynamic changes in the level of PC with decrease as the change and MPV with increase as the change along with platelet distribution width on day 1 was significantly associated with mortality. Gao et al. have shown in their study that the PC was decreased and the level of PDW increases in patients with sepsis, which could lead to an increased mortality rate [12]. Nelson and Kehl studied that the thrombocyte consumption and MPV values escalated in acute infection [13]. Increased production of platelets at the beginning of septicemia due to increased destruction and then later on myelosuppression is the cause of thrombocytopenia. Low PC explains high MPV and higher PDW. In our study, higher change in PC as seen on day 4 (after 72 hours) and after one week along with an increase in MPV after one week is significantly associated with 30-day mortality with comparable AUROCs. In case of PDW, its baseline value on day 1 was found to be significantly associated with mortality rather than its change, similar to studies conducted by Zhang et al. [5].

There could be certain confounding factors affecting the results of the study like age, high SOFA score, and comorbidities like diabetes. There were certain limitations to our study as it was conducted in a single centre at a tertiary healthcare institution with a small sample size so further prospective multicenter studies with large sample sizes are required to assess the role of these parameters in predicting mortality amongst surgical patients with sepsis.

## Conclusions

Sepsis affects the rheology of RBCs and platelets, which is responsible for changes in RDW, PC, MPV, and PDW, and also the adverse outcome in septic patients. Early identification of the changes in these parameters can help to guide early aggressive therapeutic interventions in the management of high-risk surgical patients with sepsis. From our study, we conclude that the baseline level of RDW and PDW on the day when sepsis has set in, and also the continuous decrease in PC and increase in MPV during the first week of sepsis can help predict mortality in surgical patients with sepsis. This study may provide support for further research on adding RDW, PDW, PC, and MPV to other established outcome-predicting systems and markers of mortality in patients with sepsis. In the future, both RBC and platelets may be possible targets in sepsis management, but further studies are required to improve the unfavourable outcome and mortality rate in these patients. However, there are certain limitations to the study because of the small sample size being conducted in a small centre and it did not control for other confounding factors such as comorbidities, and the severity of the illness. Moreover, the underlying mechanism behind the observed associations needs to be explored by further studies.
